# Evaluation of an HIV homecare program for lost-to-follow-up populations: a mixed methods study in Detroit, Michigan

**DOI:** 10.1186/s12981-024-00608-5

**Published:** 2024-04-12

**Authors:** L. V. Bonadonna, E. Guerrero, T. McClendon, S. Union, D. Kabbani, D. Wittmann, J. Cohn, J. Veltman

**Affiliations:** 1 Emory University School of Medicine, Atlanta, GA USA; 2 Wayne State University, Detroit, MI USA; 3 Wayne State University School of Medicine, Detroit, MI USA; 4 Wayne Health, Detroit, MI USA; 5 Loma Linda University School of Medicine, Loma Linda, CA USA

**Keywords:** HIV, Homecare, Lost-to-follow-up, Stigma reduction, Social support

## Abstract

**Background:**

Maintaining people living with HIV (PLWHIV) in clinical care is a global priority. In the Metro Detroit area of Michigan, approximately 30% of PLWHIV are out of care. To re-engage lost-to-follow-up patients, Wayne Health Infectious Disease clinic launched an innovative Homecare program in 2017. In addition to home healthcare delivery, the program included links to community resources and quarterly community meetings. We aimed to evaluate Homecare’s impact on participants’ ability to stay engaged in HIV care and reach viral suppression. We included data from PLWHIV and their healthcare workers.

**Methods:**

We used a convergent mixed-methods design, including first year program record review, semi-structured interviews, and a validated Likert scale questionnaire rating illness perception before and after Homecare. Interview data were collected from 15 PLWHIV in Metro Detroit and two healthcare workers responsible for program delivery. Semi-structured interviews focused on obstacles to clinic-based care, support networks, and illness perceptions. Interview data were transcribed and analyzed using a thematic approach. A fully coded analysis was used to create a conceptual framework of factors contributing to Homecare’s success. Means in eight categories of the Brief Illness Perception (IPQ) were compared using paired T-tests.

**Results:**

In the first year of Homecare, 28 of 34 participants (82%) became virally suppressed at least once. The program offered (1) social support and stigma reduction through strong relationships with healthcare workers, (2) removal of physical and resource barriers such as transportation, and (3) positive changes in illness perceptions. PLWHIV worked towards functional coping strategies, including improvements in emotional regulation, acceptance of their diagnosis, and more positive perspectives of control. Brief-IPQ showed significant changes in six domains before and after Homecare.

**Conclusion:**

Homecare offers an innovative system for successfully re-engaging and maintaining lost-to-follow-up PLWHIV in care. These findings have implications for HIV control efforts and could inform the development of future programs for difficult to reach populations.

**Supplementary Information:**

The online version contains supplementary material available at 10.1186/s12981-024-00608-5.

## Background

Although steady advances have been made in reducing AIDS-related deaths, efforts to reach the 90-90-90 goals defined by the Joint United National Programme on HIV/ AIDS (UNAIDS) have not moved fast enough [1]. By 2020, UNAIDS aimed to diagnose 90% of all people living with human immunodeficiency virus (PLWHIV), treat 90% of all those diagnosed with antiretroviral therapy (ART), and achieve viral suppression for 90% of people taking ART worldwide [2]. This goal has not been met, but as the world looks to achieve the 95-95-95 goals by 2030, retention in HIV care is highlighted as a major global priority for controlling disease progression and reducing mortality [3].

In the United States, there are substantial numbers of PLWHIV not maintained in clinical care [4]. In the state of Michigan, specifically, 68% of PLWHIV were retained in care and only 60% achieved viral suppression in 2020 [5]. A variety of research trials have tried to improve retention in care, including the use of peer navigators, case management, peer counseling, and/or economic incentives. Unfortunately, these programs have only had modest effect [6, 7]. Documented barriers to HIV clinical care have included poor patient-provider relationships [8], lack of social support [9], stigma [10], financial stress [11], and complex lifelong medication regimens [12] – especially in individuals with comorbid conditions [13]. There are also reports of mistrust in the healthcare system, particularly among African American communities [14].

To address gaps in the HIV care continuum, Wayne Health Infectious Disease (ID) clinic created the Homecare program to bring outpatient care to the homes of PLWHIV lost-to-follow-up in the Metro Detroit area of Michigan [15]. Launched in September 2017, Homecare focused on retention to care, and aimed to engage PLWHIV who had not visited a clinic or had HIV specific labs (CD4 or viral load) drawn in one year or longer. The program was offered to PLWHIV lost-to-follow-up at Wayne Health ID clinic and advertised to the Detroit Public Health Departments’ Data to Care Program and other community-based organizations (CBO). We conducted a convergent mixed-methods study to understand Homecare’s impact on participants’ ability to stay engaged in care and reach virologic suppression of HIV. More specifically, we aimed to investigate how Homecare engaged PLWHIV differently from clinic-based care, and if and why PLWHIV changed their perceptions of HIV during enrollment.

## Methods

### Study design

We conducted a convergent mixed methods study. We reviewed first-year Homecare Program data and then designed, collected, and analyzed qualitative interview data and quantitative survey data at the same time. The qualitative interview data and quantitative survey data represent our two data sets, embodying a convergent study design as described by Creswell [16]. This design is typically used in studies where phenomena are new or relatively unexplored [16]. We compared and merged qualitative interviews describing Homecare participants’ lived experiences with first year program data and a validated survey instrument after all individual quantitative and qualitative analyses were complete. We identified and evaluated important socioeconomic, behavioral, and structural factors mediating Homecare through: (1) semi-structured interviews and (2) a brief illness perception questionnaire.

### Interview participants

From August to December 2019, we interviewed PLWHIV participating in Homecare who willingly gave informed, written consent to participate in research. We recruited PLWHIV (*N* = 15) by convenience sampling, whereby we telephoned or messaged PLWHIV and gauged interest in participation. In three cases, we approached PLWHIV after they had visited clinic. All Homecare participants were eligible to participate, however, four did not respond, three were hospitalized, two were not able to communicate verbally, and others declined for various reasons such as lack of time, and dislike of surveys. We continued to elicit interviews until our sample size reached theoretical saturation, described in qualitative methods as the point when no new ideas emerged from the interview [17]. Subsequent data collection activities were conducted in-clinic or the PLWHIV’s home or requested location. We conducted one interview with the medical assistant and nurse practitioner responsible for delivering Homecare to elicit their perspective on program effectiveness and compare their evaluation with PLWHIV responses.

### Intervention

Once enrolled in Homecare, PLWHIV were visited by a medical assistant and nurse practitioner trained in trauma informed care and motivational interviewing. Under supervision by an ID attending physician, medical staff visited PLWHIV at their residence monthly until they reached viral suppression, defined as less than 200 HIV RNA copies in a milliliter (copies/mL) of blood. Afterwards, they were visited every three months to maintain clinical care. PLWHIV were considered “retained in care” if they completed two or more HIV medical visits in one calendar year. Homecare staff wore street clothes during home visits and arrived in an unmarked car to maintain privacy. They asked PLWHIV if they were alone prior to initiating visits, and if others in the home were aware of their HIV status. For those PLWHIV who chose to maintain complete confidentiality, Homecare staff would vocalize “your condition” instead of “HIV” during care. Additionally, staff remained careful of tone and visibility of documents and laptop while in the home. If Homecare staff encountered insect infestation or hostile environment, they would transition PLWHIV back to clinic appointments until the issue could be resolved. Some PLWHIV also requested clinic appointments if their living situation changed.

Available medical services through Homecare were comprehensive, including in-home breast, pelvic and rectal exams, blood draws, behavioral health screenings, counseling, STI treatment, immunizations, and injectable contraception. Home visits also included an environmental assessment of the home and links to CBOs for needed resources, such as houseware and other social assistance. Homecare workers had a dedicated cell phone for direct communication with patients. They used text message, video chat, and social media platforms between appointments to stay connected with patients. Homecare also provided quarterly community meetings where PLWHIV could come together to talk about shared experiences, enjoy a meal, and participate in creative activities. Homecare emphasized the role of cultural humility as a vehicle for effective social support through healthcare provider selection and training. Staff attended workshops on motivational interviewing and trauma-based care to empower PLWHIV to manage treatment with ART. To evaluate the effectiveness of the Homecare program, we performed a convergent mixed-methods study from August to December 2019.

### Data collection

#### Quantitative data collection

We reviewed first year program records with data from all PLWHIV enrolled in Homecare from September 2017 to September 2018 to evaluate retention to care and viral suppression. Afterwards and separately, we surveyed 15 PLWHIV using the Brief Illness Perception Questionnaire (IPQ) during qualitative interviews between August and December 2019. Validated by Broadbent et al. [18], the Brief-IPQ allowed us to measure cognitive representations of illness perceptions before and after participation in the HIV Homecare program. The eight-item questionnaire employs a 10-point Likert scale to measure domains of consequence, timeline, personal control, treatment control, identity, concern, understanding, and emotional response to living with HIV. A choice of 0 indicated the lowest perception of each domain, and a score of 10 indicated the highest. At the time of the interview, we asked PLWHIV to rank their perceptions before Homecare, and then asked them to rank their current perceptions.

#### Qualitative data collection

All PLWHIV were interviewed using a semi-structured interview guide, focusing on themes of (a) social supports/network, (b) economic environment, (c) trust in the healthcare system, and (d) motivation and personal agency. We focused on themes identified as key modifiers for retention to care by these health professionals, and supported by literature review [8–11]. We explored the differences between clinic-based care and Homecare by specifically prompting PLWHIV to describe their experiences in both systems. Our goal was to collect rich descriptions of how Homecare changes the experience and availability of biomedical and psychosocial care in comparison to a traditional clinic model. The interview guide was developed in collaboration with three health professionals with 30 years combined experience working with PLWHIV. The guide was piloted with one PLWHIV and then modified based on their responses. The final interview guide can be found in the appendix. All interviewers were conducted by the first author. Interviews lasted 14 to 58 min, with a median time of 35 min. Results of the pilot interview are included here. To triangulate data sources, we also interviewed Homecare program staff.

#### Analysis

All interviews were recorded, transcribed, and uploaded into Atlas.ti qualitative data analysis software. We conducted a data-driven thematic analysis, as described by Braun et al. [19]. We proceeded inductively, reading and re-reading transcripts for deep familiarization, developing a codebook, and then applying codes to transcripts, making frequent comparisons between texts [19]. All transcripts were coded by the first author, and approximately 40% were independently coded by the fourth author, reviewed, and discussed for reliability. After revising codes, and reaching consensus, we grouped codes into three categories of factors influencing Homecare’s impact on PLWHIV and created a conceptual framework to display theoretical insights. Table 1 shows the codes we used to build each category. All quantitative analyses were performed using Microsoft Excel. We summarized continuous data by medians and interquartile ranges (IQR). We compared results from the Brief-IPQ before and after Homecare using paired T-tests. All p-values generated were two sided, with significance set at 5%. We integrated qualitative and quantitative data through a Joint Display Analysis showing box plots of the Brief-IPQ with representative quotes of each questionnaire domain shown beneath. We also used the Brief-IPQ to support the development of the conceptual framework of Homecare’s impact on retention in HIV care.

**Table 1:** Conceptual codes

Contributing Codes		
**Changing Perceptions of Illness**	**Removing Physical Barriers**	**Social Support and Stigma Reduction**
Concern of HIV Homecare – emotional support Homecare – relationship with staff Knowledge Support Knowledge support – examples Medications Personal Control Symptoms	Clinic – obstacles to care Homecare – structure and location	Clinic – obstacles to care Clinic – relationship Homecare – relationship with staff Homecare – structure and location Reaction to Diagnosis Reaction from family/peers Social Support and Network Stigma

## Results

### Quantitative results

In its first year, Homecare enrolled 34 PLWHIV previously lost-to-follow-up in clinic-based care. The majority identified as African American (*n* = 31) and male (*n* = 26). After one year of enrollment, 31 were retained in care with at least 2 medical visits. Additionally, 28 Homecare participants achieved viral loads < 200 copies/mL at least once during fifteen months of follow up.

Table 2 displays interview participants’ demographic characteristics and their perceived barriers to care. The participants’ median age was 42 and 80% were male. Participants had been living with HIV for a median of 10 years and had been in Homecare for a median of 631 days.

**Table 2:** General Characteristics of Interview Participants enrolled in Homecare

Characteristics	Units	Interview Participants (*N* = 15)
**General**		
Age	Median (IQR)	42.3 (31–52)
Male	*n* (%)	12 (80)
African American	*n* (%)	15 (100)
Number of Years Living with HIV	Median (IQR)	10 (6–18)
Number of Days in Homecare	Median (IQR)	631 (460–702)
**Physical Barriers to Clinic-Based Care**		
Lack of transportation	*n* (%)	11 (73)
Work Obligations	*n* (%)	6 (40)
Childcare responsibilities	*n* (%)	2 (13)

Results from eight categories of the Brief-IPQ before and after participation in Homecare are shown in Table 3. Means were significantly decreased (all *p* < 0.05) for the domains: emotional response, consequences and identity. PLWHIV reported HIV affecting them less emotionally, having less impact on their daily life, and experiencing fewer health sequelae after being enrolled in Homecare. Means were significantly increased (all *p* < 0.005) for the domains: personal control, treatment control, and understanding of HIV. PLWHIV reported more personal control over their HIV treatment, greater appreciation for the benefits of treatment, and better understanding of HIV in general after Homecare.

The domains timeline and concern did not show statistically significant results. Almost every PLWHIV always knew that HIV was a life-long diagnosis that could not be cured. Concern over HIV status was variable – some PLWHIV coped through repression before Homecare and grew more concerned of HIV as they began to acknowledge, accept, and appreciate treatment management; and others became less concerned through Homecare because they gained a better understanding of their health status and concrete steps to becoming virally suppressed.

**Table 3:** Brief Illness Perception Questionnaire

Illness Perception	Mean 0–10 Likert Scale		*P*-value
	Before Homecare	Currently	
Timeline (*n* = 14)	9.5	8.7	0.19
Concern (*n* = 15)	6.5	4.8	0.23
Emotional Response (*n* = 15)	7.9	3.3	< 0.00*
Consequences (*n* = 15)	6.9	3.8	0.01*
Identity (*n* = 15)	4.4	2.1	0.04*
Personal Control (*n* = 15)	4	8.4	< 0.00*
Treatment Control (*n* = 15)	5.4	9.3	< 0.00*
Understanding (*n* = 15)	6	9.3	< 0.00*

*Statistical significance at *P* ≤ 0.05

0 indicates lowest perception, 10 indicates highest. See Appendix 1 for B-IPQ

### Qualitative results – interview findings

**Fig. 1:**
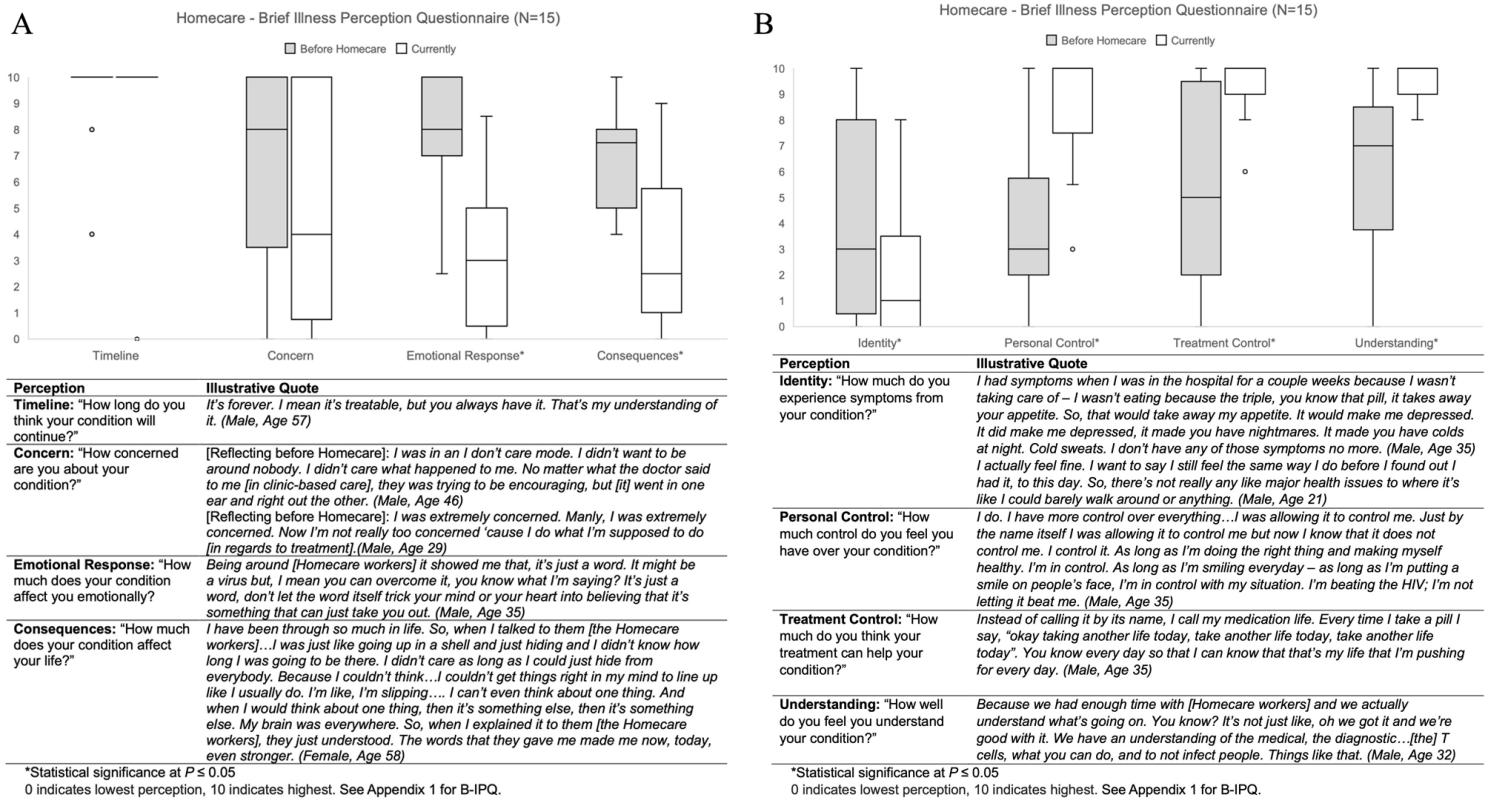
**A** and **B**: Joint display. Illness perceptions before and after enrollment in Homecare are presented in box plots, showing median (central line), interquartile range (box), range (whiskers), and outliers (open circles). Quotes displayed below illustrate Homecare participants’ explanation for changing perceptions. 0 indicates lowest perception, 10 indicates highest. See Appendix 1 for B-IPQ

Interview findings were organized conceptually into three categories: (1) social support and stigma reduction, (2) removing physical and resource barriers, and (3) changing perceptions of illness. Categories one and two are represented below with quotes from PLWHIV and their healthcare workers. The third category is incorporated in the mixed methods results, with interview findings presented in Fig. 1.

#### Social support and stigma reduction

Upon receiving diagnosis of HIV, many PLWHIV enrolled in Homecare experienced some form of rejection from family and/or peers.

*“There wasn’t anybody I could talk to. I tried to tell my family and they ostracized me.” (PLWHIV, Male, Age 71)*.

Some chose not to disclose their status to anyone or to only very select people in their life for fear of causing their loved ones excessive worry or burden.

*“I haven’t gotten to the point where I feel comfortable with exposing it because I haven’t fully 100% accepted it.… I don’t want my children to get something to worry about, give my ex-wife something to worry about. That’s just between me and God…. I don’t want that to be something that someone else has to go through…. That’s my actions, that’s my results…. So, nobody else has to share that debt but me.” (PLWHIV, Male, Age 46)*.

This limited or nonexistent support network meant that PLWHIV were entirely responsible for enrolling, navigating, and maintaining their healthcare. Additionally, PLWHIV described feelings of shame associated with their status as they faced challenges with perceived stigma which made them want to push others away.

*“I still felt like an outcast because…people try to hide it, but I see it…. This is something I have to deal with…. So, I just started using it as a weapon to keep people away…. People when they come up to me, especially guys, the first thing that come out my mouth, ‘I got HIV’…. It’s a defense mechanism for me. It just keeps a lot of people away.” (PLWHIV, Male, Age 46)*.

Building relationships with healthcare providers was often complicated by a lack of continuity of care, and the strain of having to repeatedly explain their social and medical histories to new providers.

*“When I was in the clinic, I had probably seen about four different doctors. I had to keep – it was like ‘so, what happened, what happened’ – I had to keep talking about a traumatic experience over and over and over!” (PLWHIV, Male, Age 21)*.

Interactions with healthcare staff were, for many, one of the very few or only outlets PLWHIV had to discuss their HIV status openly. Some PLWHIV interviewed had good working relationships with their clinic-based providers, but others had difficulty forming a trusting bond.

*“I had several doctors that I just did not like. They had poor bedside manner. It was just like a matter a fact thing for them. Like, dude I think I’m dying. You know one of those ‘should’ve-known-better’ attitudes.” (PLWHIV, Male, Age 57)*.

In contrast to clinic-based care, relationships with Homecare workers were perceived as consistent and widely praised as uplifting, warm, and non-judgmental.

*“I just felt like it was a routine [at the clinic]…. Just another guy with HIV. With them [Homecare workers], they make you feel a little more like they understand. They can talk to you. It’s about having that one on one, not being the next one.” (PLWHIV, Male, Age 46)*.

Homecare workers were seen by participants as helping them focus on self-reflection and life goals not only for their health, but for their future as well.

*“I took it a lot more serious, ‘cause it was a lot of conversation, not just a whole bunch of medical conversation. It was conversation about my life. Um, what do I want to do in the future. So having these types of conversations of what I want to do in the future, I had to be healthy to do these things.” (PLWHIV, Male, Age 29)*.

The quarterly community meetings, where individuals in the program could gather for food, conversation, and a guest speaker, also contributed to this sense of shared experience, community, and support:

*“It just keeps you motivated to, like, help other people. Encourage other people. When you sit around these people. You build relationships. I still talk to a few of the guys outside of it. And…you need a support system outside of it.” (PLWHIV, Male, Age 29)*.

Homecare workers discussed the role of cultural humility in the program, and the need for empathy, understanding, and shared experience to foster strong relationships with PLWHIV.

*“When people are even thinking about starting a program like this you have to consider the population that you’re dealing with. You have to get people who are culturally sensitive to that population, people who may be accepted….” (Homecare worker, Female, Age 43)*.

There were negative opinions shared by three PLWHIV interviewed. Two individuals were disappointed there were not more opportunities for peer interaction through the Homecare program. One individual was disappointed with the length of time between appointments after he achieved viral suppression and hoped to see Homecare staff more frequently.

#### Removing physical and resource barriers

Lack of transportation was a barrier to care for 73% of PLWHIV. Additionally, 40% noted work obligations, and 13% reported childcare responsibilities as other barriers to care. Most PLWHIV interviewed could arrange for medical transportation services to their clinic appointment, but many described these services as unreliable.

*“You have the medical transportation, you know. Sometimes they come, sometimes they don’t. Or sometimes they come after your appointment time….” (PLWHIV, Male, Age 41)*.

Entering the clinic itself was a barrier, as worries about privacy and confidentiality were troubling for many PLWHIV. This was compounded by the fact that wait times were often long, and PLWHIV were left sitting in open spaces.

*“Before I was always paranoid when I would go to the clinic. Who will see me? And if I see someone, I know I’ll try to hide and be isolated or I’d constantly be going to the bathroom….I remember telling the lady at the counter once, when y’all ready for me can you just call me on my cell phone. Don’t announce my name.” (PLWHIV, Male, Age 33)*.

In contrast, Homecare workers were described as punctual; they came directly to the PLWHIV’s home – eliminating several barriers described above.

*“They [Homecare workers] come to my house. They’re ready to go. If I go to the clinic… when I get there, they’re ready to see me or I might have to wait…. You know, a reasonable amount of time [would be] 5–10 minutes, but an hour? Come on…” (PLWHIV, Male, Age 57)*.

Homecare providers commented on the value of additional time for environmental home assessments to connect PLWHIV to community resources. As described by a Homecare worker:

“*For a new patient [an appointment could] be like an hour and a half to two hours because you’re not just in there for the patient assessment. You’re in there doing a whole environmental assessment also because do they have water, do they have heat, do they have a bed, do they have the necessities that they need because that’s where I touch base with the community organizations, request a bed, request a refrigerator…” (Homecare worker, Female, Age 49)*.

### Mixed methods results

**Fig. 2:**
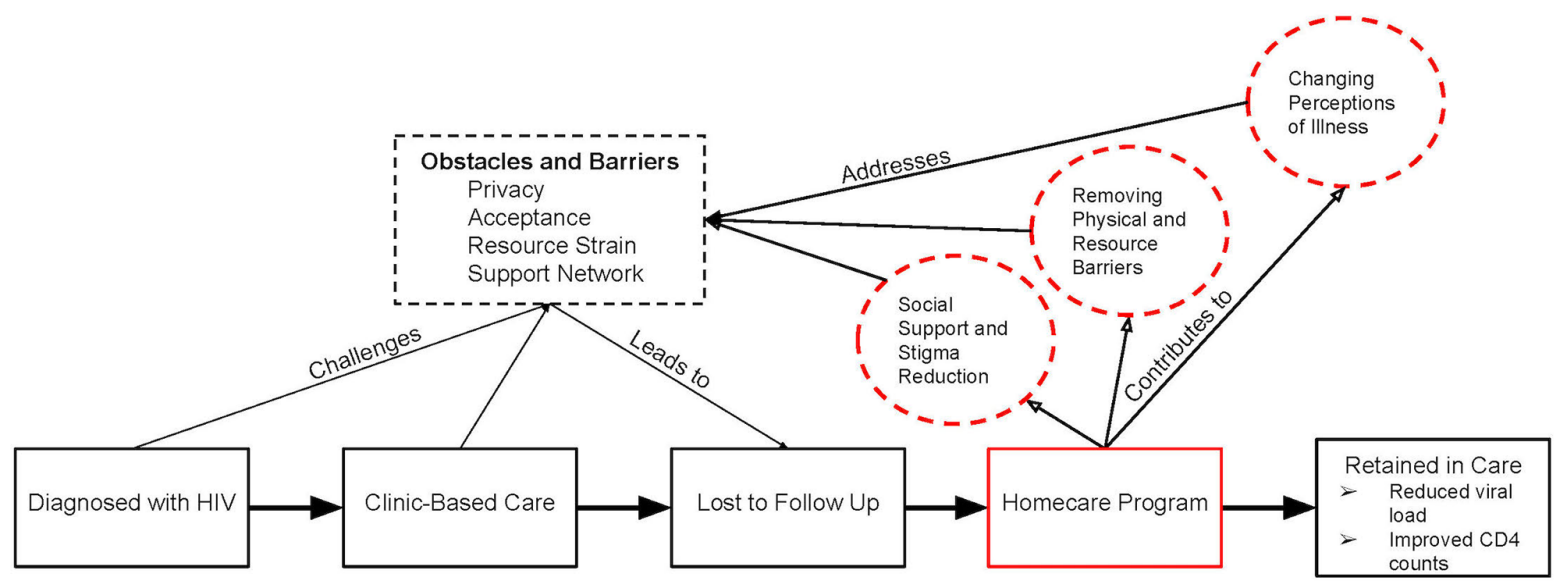
Conceptual framework. Based on qualitative and quantitative data presented in the results section, Homecare mediates return to and maintenance in care for lost-to-follow-up people living with HIV

Figure 1 A and Fig. 1B show a joint display analysis of the Brief-IPQ, with box plots displaying Likert scale results and representative quotations for each domain. We included the exact questions we asked PLWHIV and their responses. Both quantitative and qualitative findings provided evidence that participation in Homecare improved PLWHIV’s ability to reach virologic suppression, remain in care, and positively impact perceptions of illness, including emotional responses to HIV. Additionally, the impact of HIV on daily functioning was significantly reduced, including reductions in negative health consequences. This is shown through direct quotation and Brief-IPQ results. Through Homecare, PLWHIV changed their framework of control, shifting to a person-centered approach to managing their healthcare plans. PLWHIV improved their understanding of the scientific intricacies of HIV care, such as medication resistance and markers of immune function. Moreover, they accepted their treatment’s vital role for their physical health and survivorship. The conceptual framework in Fig. 2 displays obstacles and barriers that contribute to PLWHIV becoming lost-to-follow-up and how the Homecare program mediates their return to care. Quantitative and qualitative data were complimentary, showing significant results in the Brief-IPQ with concordant direct interview quotations, and both were considered to build this framework.

## Discussion

Retaining PLWHIV in clinical care is a major global priority for reducing morbidity and transmission of HIV [2]. Our results show that Homecare can help PLWHIV remain in care and achieve viral suppression through (1) improved social support and stigma reduction, (2) removal of physical and resource barriers to care and (3) changed perceptions of illness. Understanding these three pillars may help strengthen health systems seeking to improve treatment adherence and viral suppression strategies.

Both qualitative and quantitative studies have repeatedly shown that stigma and discrimination impact people’s decision to access treatment for HIV [20]. In a systematic review of these effects across cultural contexts, there were multiple levels of influence, including intrapersonal, interpersonal, and structural stigmas enacted on PLWHIV [10]. We found participants in the current study faced these same circumstances – shame of their diagnosis, concealment, rejection from family, and, in some cases, judgement from clinic-based providers. This compromised available social support, and by extension, adaptive coping strategies. Enrollment in Homecare, however, grounded PLWHIV in trusting relationships with healthcare providers and provided a space for quarterly community meetings with other PLWHIV. The only negative opinions of the program shared were from individuals who hoped from more time with peers and Homecare staff, which speaks to the importance of these connections. The home-setting allowed people to open up and form closer bonds with their healthcare providers, without a sense of time-pressure, or uneasiness with the setting. Homecare staff were discreet and prioritized confidentiality during home visits. Strong patient-provider relationships and available social support have been documented as facilitators to medication adherence in different settings [8, 21, 22]. Serving a largely African American community, Homecare providers remained vigilant of racial disparities in HIV, and reported an understanding of unique barriers facing their communities. Other studies have shown that cultural competency significantly affects HIV care, especially among minority groups, in Detroit and other American cities [23–25]. Considering African Americans continue to face the highest burden of new HIV infections compared to other racial/ ethnic groups in the United States, Homecare exemplifies how cultural inclusivity can contribute to viral suppression in this group [26].

Like other studies, we found that many PLWHIV faced financial hardship, lack of transportation and work obligations that prevented them from attending clinic appointments [11, 27]. To mitigate economic barriers to care, some research trials have offered conditional economic incentives to improve HIV treatment adherence. In the United States, this showed only modest effect in short-term adherence rates in a large, multi-city community based trial [7]. When evaluating long-term adherence, other studies have shown no significant gains in treatment adherence after the active intervention period [28]. Homecare strategized away from economic incentives, and instead focused on eliminating resource barriers through increased time, punctuality, and convenience with healthcare provider visits. In attempts to alleviate financial burdens, Homecare workers used the current resource network around Metro Detroit to connect PLWHIV with existing social services and benefits programs. Transportation problems were eliminated because Homecare workers came to PLWHIV’s residence and offered flexible scheduling and frequent appointment reminders. Worry about being “found-out” by physically entering a clinic associated with HIV was eliminated through Homecare. Concerns of confidentiality and unintended disclosure have been demonstrated as a barrier to care in other settings as well [29, 30].

PLWHIV in our study showed significant changes in six domains of the Brief-IPQ. These illness perceptions were first described by Leventhal et al. as dynamic processes by which people attempt to understand their illness and then adjust their behavior to cope with health threats [31]. Our study showed that after participation in Homecare, PLWHIV changed the role of HIV in their life. With the help of Homecare workers, they worked towards functional coping strategies, including improvements in emotional regulation, acceptance of their diagnosis, and shifting perspectives of the control and power they possess. In accordance with our work, a multi-site, cross cultural study found that lower perceptions of consequence and greater controllability may improve PLWHIVIV’s ability to cope with illness-related stressors [32]. Additionally, other studies have shown that the perception of greater consequences on one’s life and greater emotional impact were related to higher viral loads [33, 34]. This may highlight the way in which Homecare’s impact on illness perceptions led to viral suppression for many of the participants. Many Homecare participants also reported improvements in their understanding of HIV, associated symptoms, their medications and treatment plans.

There are several limitations to our study. Reporting and recall bias may have occurred because we asked PLWHIV to comment on events that occurred in the past, including ranking the effect HIV had on their life before Homecare. Additionally, selection bias may have occurred because we used convenience sampling to select participants. This is less likely, however, because most people we asked agreed to participate in the study. All study participants were offered a $25 gift card for their time, which may have influenced their interview responses. We tried to eliminate this by emphasizing that participation in the study would not affect clinical care, and all participants had the option of ending the interview at any time. The interview transcripts were fully coded by the first author and partially coded by the fourth author for data reliability purposes. Coders remained aware of their preconceived ideas and biases and used bracketing, described in qualitative methods [35], to prevent these ideas from influencing data analysis. Data were collected until the point of saturation when no new ideas emerged from interviews.

Future research might investigate the transition from Homecare program back to clinic-based care. Longitudinal analysis of viral load suppression is needed to evaluate the effectiveness of Homecare. Additionally, cost-effectiveness analysis may help show how Homecare compares to other clinical adherence interventions, including those with conditional and unconditional economic incentives. Reproducing and evaluating Homecare in other settings with a greater sample size may help improve reliability of the results.

## Conclusion

Overall, this study demonstrated that it is possible to re-engage lost-to-follow-up PLWHIV, retain them in care, and reach viral suppression. Through work grounded in cultural humility, Homecare improved social support, reduced stigma, removed resource barriers, and helped change illness perceptions in PLWHIV in Detroit. These findings have significant implications for HIV control efforts and provide evidence for including lost-to-follow-up PLWHIV in consideration for new long-acting injectable HIV medications. These results could inform the development of future programs for difficult to reach populations.

### Electronic supplementary material

Below is the link to the electronic supplementary material.

Supplementary Material 1

## Data Availability

All available data is included within this published article. The S1 Appendix contains interview guides for people living with HIV and healthcare personnel.

## List of Abbreviations

PLWHIV: People Living with HIV
UNAIDS: United National Programme on HIV/ AIDS
ART: Antiretroviral therapy
ID: Infectious Disease
CBO: Community-based organizations
IPQ: Illness Perception Questionnaire
IQR: Interquartile ranges
