# Supplementary material for: Evaluation of an HIV homecare program for lost-to-follow-up populations: a mixed methods study in Detroit, Michigan
Source: AIDS Res Ther. 2024 Apr 12;21:21. doi: 10.1186/s12981-024-00608-5 (PMC11015688; doi:10.1186/s12981-024-00608-5)
Supplement: Supplementary file 1 — Supplementary Material 1 [file 12981_2024_608_MOESM1_ESM.docx]

**Appendix 1 – HIV Homecare: Person Living with HIV Interview Guide**

**Participant ID: ___________ DOB: _____________**

**General**

| Date of Interview: | / / |
| --- | --- |
| Location of Interview: |  |
| Interviewer: |  |

**Dates of Service**

| Diagnosis of HIV | / / |
| --- | --- |
| Initiation of Homecare | / / |

**The Brief Illness Perception Questionnaire**

1. How much does your condition affect your life?

**Before Homecare**

0 1 2 3 4 5 6 7 8 9 10

No affect at all Severely affects my life

**Currently**

0 1 2 3 4 5 6 7 8 9 10

No affect at all Severely affects my life

1. How long do you think your condition will continue?

**Before Homecare**

0 1 2 3 4 5 6 7 8 9 10

A very short time forever

**Currently**

0 1 2 3 4 5 6 7 8 9 10

A very short time forever

1. How much control do you feel you have over your condition?

**Before Homecare**

0 1 2 3 4 5 6 7 8 9 10

Absolutely no control Extreme amount of control

**Currently**

0 1 2 3 4 5 6 7 8 9 10

No affect at all Extreme amount of control

1. How much do you think your treatment can help your condition?

**Before Homecare**

0 1 2 3 4 5 6 7 8 9 10

Not at all Extremely helpful

**Currently**

0 1 2 3 4 5 6 7 8 9 10

Not at all Extremely helpful

1. How much do you experience symptoms from your condition?

**Before Homecare**

0 1 2 3 4 5 6 7 8 9 10

No symptoms at all Many severe symptoms

**Currently**

0 1 2 3 4 5 6 7 8 9 10

No symptoms at all Many severe symptoms

1. Before and after starting Homecare, how concerned are you about your condition?

**Before Homecare**

0 1 2 3 4 5 6 7 8 9 10

Not at all concerned Extremely concerned

**Currently**

0 1 2 3 4 5 6 7 8 9 10

Not at all concerned Extremely concerned

1. Before and after starting Homecare, how well do you feel you understand your condition?

**Before Homecare**

0 1 2 3 4 5 6 7 8 9 10

Don’t understand at all Understand very clearly

**Currently**

0 1 2 3 4 5 6 7 8 9 10

Don’t understand at all

Understand very clearly

1. Before and after starting Homecare, how much does your condition affect you emotionally? (e.g. does it make you angry, scared, upset or depressed?

**Before Homecare**

0 1 2 3 4 5 6 7 8 9 10

Not at all affected emotionally Extremely affected emotionally

**Currently**

0 1 2 3 4 5 6 7 8 9 10

Not at all affected emotionally Extremely affected emotionally

*Part 1: Introduction*

**This guide represents the main themes to be discussed with participants. Non-leading and general prompts may also be used, such as, “Can you tell more about that.”*

1. **Establishing Rapport:** Before we begin, could you tell me a little about yourself? How long have you been living in this neighborhood?
2. When were you first diagnosed with HIV? ____/____/_____
3. Tell me about your experience starting treatment.
   1. Prompt: were you given any resources to help you learn about HIV? Were they helpful?
   2. Prompt: How was it understanding the medication regimes?
   3. Prompt: What were your biggest concerns at the time? (Example: side-effects to the medication, employment issues, sadness/depression, personal relationships, etc.)
   4. Prompt: Discuss results from BIPQ question #1

*Part 2: Social Support/ Networks*

1. Since diagnosis, have you revealed your HIV status to other people in your life? How have they reacted?
   1. Prompt for description of social support networks.
   2. Prompt: Have you ever felt like you had to hide your diagnosis?
2. When you were in clinic-based care, do you feel like you had people that encouraged you to go to your medical appointments?

*Part 3: Economic Environment*

1. When you were receiving treatment in clinic, did you ever have to miss an appointment because of work or school obligations? No  Yes
2. Has the cost or reliability of transportation ever been an issue in receiving treatment?

No  Yes

1. Are you a caregiver for any children? No  Yes
2. Has the homecare program helped you overcome any work, transportation, or childcare barriers to receiving treatment?
   1. Prompt: Do you feel like you have more time now that you are in the homecare program?

*Part 4: Trust in the healthcare system*

1. When you were receiving treatment in-clinic, can you describe your relationship with staff?
   1. Prompt: How did the staff make you feel?
   2. Prompt if the staff ever made them feel judged/ stigmatized/ welcome/supported
   3. Prompt: Did you trust the staff in clinic?
   4. Prompt if they have ever missed an appointment because of stigma from health providers.
2. How is your relationship with the homecare staff different, if at all?
   1. Prompt if homecare staff make them feel welcome and supported.
   2. Prompt if homecare staff has ever made them feel stigmatized.
   3. Prompt: What do you like about the homecare staff? (Example: quality of care they provide, relatability, communication abilities)
   4. Prompt: Have the homecare staff helped you deal with any feelings of stigma?
   5. Prompt: Discuss results from BIPQ question #8

*Part 5: Motivation and Agency*

1. Apart from what we’ve already discussed, are there any other reasons why you stopped receiving treatment in-clinic?
2. Before and after being in the Homecare program, how much control do you feel you have over your illness?
   1. Prompt: Discuss results from BIPQ question #3
3. How would you describe your understanding of HIV and treatment before and after starting homecare?
   1. Prompt: Discuss results from BIPQ question #2,4,6,7
4. How would you describe your health before and after starting homecare?
   1. Prompt: Discuss results from BIPQ #5

*Part 6: Conclusion*

1. Are there any improvements that you think could make the homecare program better?

Prompt: Is there anything you dislike about the homecare program?

**Appendix 3 – HIV Homecare: Healthcare Personnel Interview Guide**

| Date of Interview: | / / | |
| --- | --- | --- |
| Position of Healthcare worker: |  | |
| Interviewer: |  | |
| Participant ID: |  | DOB: / / |

**This guide represents the main themes to be discussed with participants. Non-leading and general prompts may also be used, such as, “Can you tell more about that.”*

1. Could you tell me about your background working with PLWH?
2. For the people enrolled in homecare, what are some of the common challenges or obstacles they experience during clinic-based care?
   1. Prompt for economic challenges such as transportation, finance, and childcare responsibilities.
3. How would you describe the support networks of people participating in homecare?
4. In general, how do people respond to you when they first start homecare?
5. How do you think your relationship with homecare participants has developed over time?
   1. Prompt: How would you describe your relationship with PLWH in the homecare program, now? Do you believe your relationship with PLWH is different than the relationship they had with their healthcare workers in clinic-based care? What do you think accounts for the difference (if any)?
   2. Prompt: How do you think the homecare program has helped people overcome these obstacles?
6. Since starting homecare, what changes have you noticed in participant’s attitudes and understanding of HIV?
   1. Prompt: What do you think has caused these changes?
   2. Prompt for changes in motivation, and sense of agency and control of their health.
7. What resources do you think will help participants be successful in clinic-based care in the future?
8. Last year, the homecare program had the highest percentage of virally unsuppressed individuals achieve viral suppression in Michigan. Apart from what we’ve already discussed, why do you think this program has been so successful?
9. Are there any improvements that you think could make the homecare program even better?
